# MarFERReT, an open-source, version-controlled reference library of marine microbial eukaryote functional genes

**DOI:** 10.1038/s41597-023-02842-4

**Published:** 2023-12-21

**Authors:** R. D. Groussman, S. Blaskowski, S. N. Coesel, E. V. Armbrust

**Affiliations:** 1 School of Oceanography, University of Washington, Benjamin Hall IRB, Room 306 616 NE Northlake Place, Seattle, WA 98105 USA; 2 Molecular Engineering and Sciences Institute, University of Washington, Molecular Engineering & Sciences Building 3946 W Stevens Way NE, Seattle, WA 98195 USA

**Keywords:** Classification and taxonomy, Microbial genetics, Transcriptomics

## Abstract

Metatranscriptomics generates large volumes of sequence data about transcribed genes in natural environments. Taxonomic annotation of these datasets depends on availability of curated reference sequences. For marine microbial eukaryotes, current reference libraries are limited by gaps in sequenced organism diversity and barriers to updating libraries with new sequence data, resulting in taxonomic annotation of about half of eukaryotic environmental transcripts. Here, we introduce Marine Functional EukaRyotic Reference Taxa (MarFERReT), a marine microbial eukaryotic sequence library designed for use with taxonomic annotation of eukaryotic metatranscriptomes. We gathered 902 publicly accessible marine eukaryote genomes and transcriptomes and assessed their sequence quality and cross-contamination issues, selecting 800 validated entries for inclusion in MarFERReT. Version 1.1 of MarFERReT contains reference sequences from 800 marine eukaryotic genomes and transcriptomes, covering 453 species- and strain-level taxa, totaling nearly 28 million protein sequences with associated NCBI and PR^2^ Taxonomy identifiers and Pfam functional annotations. The MarFERReT project repository hosts containerized build scripts, documentation on installation and use case examples, and information on new versions of MarFERReT.

## Background & Summary

Microbial eukaryotes perform essential ecological functions in marine ecosystems as phototrophs, predators, and parasites^1^. This evolutionarily diverse group of organisms collectively possesses hundreds of millions of taxonomically distinct genes encoding metabolic processes that shape global biogeochemical cycles^2^. Eukaryotic metatranscriptomes are a sample of the nucleotides that result from community-wide transcriptional patterns and provide a window into how different members of the community function *in situ*. Metatranscriptome samples have been collected and annotated for studies of marine microbial eukaryotes across the world, including the global-scale *Tara* Oceans expedition^2^. Identifying the taxonomic origin of these sequences depends on the quality and depth of the reference sequence library used for annotation. Early metatranscriptome analyses were limited by sparsity of reference sequences among marine protists, with many lineages lacking any sequenced representatives. The sequence landscape improved dramatically in 2014 with development of the Marine Microbial Eukaryote Transcriptome Sequencing Project (MMETSP), a community-wide undertaking that resulted in public availability of 678 assembled transcriptomes derived from over 340 marine eukaryotic strains^3^. The MMETSP increased the number of sequenced marine protist species by approximately one order of magnitude, greatly enhancing the breadth and quality of environmental sequence annotation.

Since the release of the MMETSP, a variety of new marine eukaryote-focused reference libraries have arisen that build upon MMETSP by aggregating additional marine microbial genomes and transcriptomes from different sources (Table 1). The PhyloDB reference library^4^ was last updated publicly in 2015 and contains microbial eukaryotes, bacteria, archaea, and viruses isolated from both marine and non-marine environments. MARMICRODB^5^ is a prokaryote-focused database that includes a subset of eukaryotic reference sequences from the MMETSP. The EukZoo protein database^6^ of aquatic microbial eukaryotes was released and updated in 2018 and added 61 genomes and transcriptomes to the MMETSP dataset. The METdb repository^7^ was assembled in 2019 and included MMETSP re-assemblies^8^ in addition to 34 marine protist transcriptomes generated from cultures within the Roscoff Culture Collection (RCC). Most recently, the EukProt database^9^ was released in 2022 and incorporates genome-scale predicted proteins from a broad spectrum of eukaryotic phyla, including a high proportion of sequences from terrestrial plants, animals, and fungi along with 272 of the 678 MMETSP transcriptomes. These culture-driven sequencing and database efforts have contributed significantly to the landscape of available reference sequences, but coverage gaps in taxonomic representation persist, and approximately half of assembled marine metatranscriptome transcripts from the *Tara* Oceans expedition and other environmental sequencing studies have no significant similarity to any known sequence in reference sequences libraries^2,10^.

**Table 1:** Comparison of reference sequence library features.

Library Name	Latest Release	Total Entries	Domain Focus	Reviewed Publication	Code	Data DOI
MMETSP^3^	2014	678 transcriptomes	Marine microbial eukaryotes	Yes	No	No
PhyloDB^4^	2015	19,962 viral, 230 archaeal, 4910 bacterial, 894 eukaryotic taxa (409 from MMETSP)	General	No	No	No
EukZoo^6^	2018	739 genomes, transcriptomes (678 from MMETSP)	Aquatic microbial eukaryotes	No	Yes	Yes
METdb^7^	2019	464 transcriptomes (410 from MMETSP)	Marine microbial eukaryotes	Poster	Yes	No
MMETSP re-assemblies^8^	2019	678 MMETSP transcriptomes	Marine microbial eukaryotes	Yes	Yes	Yes^17^
EukProt^9^	2022	993 species (272 from MMETSP)	General eukaryotes	Yes	No	Yes
MarFERReT	2023	902 total genomes & transcriptomes (678 from MMETSP), 800 QC-validated entries	Marine microbial eukaryotes	Yes	Yes^299^	Yes^298^

Library Name: shorthand name of the database. Latest Release: most recent release of the database or publication. Total Entries: number of included references, broken down by provenance. Domain Focus: taxonomic emphasis of the database. Publication: peer-reviewed publication accompanies the database. Code: availability of an accessible codebase complete with documentation.

The emergence of single-cell amplified genomes and transcriptomes (SAGs and SATs) now allows for targeted sequencing of single cells from environmental samples without the need for culturing^11^, opening the window of sequencing opportunities for the uncultured majority of marine eukaryote species. Taxonomic identification of a eukaryotic SAG is assigned, when possible, from analysis of 18 S rDNA generated during the amplification step, although as with other annotations, 18 S rDNA-based taxonomy is also dependent on reference sequences. SAGs from MAST-3 and MAST-4 clades of marine stramenopiles and the Chrysophyte H1 and H2 clades are publicly available^12^. The availability of SAGs from uncultured organisms in combination with the continued isolation and sequencing of marine eukaryote strains from diverse environments such as Antarctic coastal waters^13^, deep waters^14^, and the oligotrophic open ocean^15^ improves the ability to provide taxonomic affiliation to previously unannotated sequences.

As additional cultured and uncultured reference transcriptomes and genomes from different sources continue to become publicly available, their ongoing integration into open and reproducible reference sequence libraries remains vital to enhancing environmental sequence annotation. Alongside the static database release, publishing documented code for generating the database product ensures reproducibility and transparency in the release of future library versions. Furthermore, open-source development of a library codebase allows researchers to expand the core codebase and dependencies and fork the codebase independently as desired, thus helping to ensure the resource continues to grow. This also allows researchers to add new reference sequences for targeted research questions without dependence on centralized library releases. Reference libraries currently available for marine microbes either do not meet these criteria (Table 1) or they lack the necessary strain- or species-level diversity to annotate marine microbial eukaryote metatranscriptomes. For instance, the objective of the recently released EukProt^9^ reference database is designed to include at least one representative genera from sequenced lineages for broad eukaryotic diversity, and leave out sequenced species in well-sequenced lineages to balance the total database size. Although useful for broad annotation purposes, this does not meet the specific research needs of metatranscriptome studies that investigate species- and strain-level sequence differences in diverse marine eukaryote lineages^15,16^.

Here, we introduce the Marine Functional EukaRyotic Reference Taxa (MarFERReT), an updated open-source marine eukaryote reference protein sequence library with a reproducible framework allowing for community-supported expansion over time. MarFERReT was created out of the need for a reference sequence library that 1) focuses on species and strain-level representation of marine microbial eukaryotes, 2) is represented by a stable and accessible publication and/or DOI, 3) captures recent advances in published sequence data, 4) has transparent and replicable code, and 5) can expand over time with documented releases. MarFERReT v1 was designed to be a comprehensive marine microbial eukaryote reference library for the taxonomic annotation of environmental metatranscriptome data that facilitates access to marine eukaryote reference sequences and helps further our understanding of the diverse functional potential of marine protists.

**Fig. 1:**
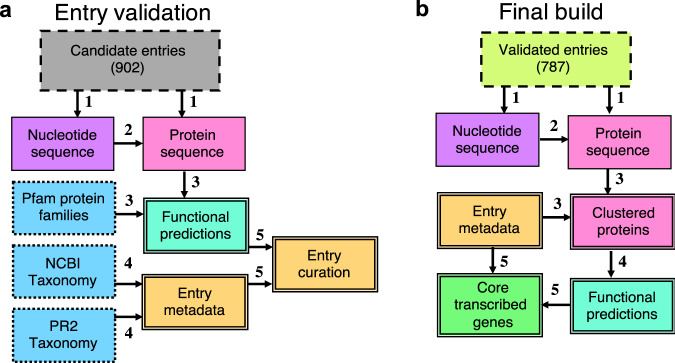
Diagrammatic overview of MarFERReT validation and build processes. Boxes represent the data sets involved in building MarFERReT and the border style indicates the data type: external sequence inputs (dashed line), external taxonomic and functional annotation resources (dotted lines), internal data products (single solid line) and output MarFERReT data products (double lines). Arrows indicate processes. (**a**) Candidate entry and NCBI taxID validation: (1) Candidate entries were identified from primary data sources and downloaded as nucleotide and protein reference sequences; (2) six-frame translation^301^ and frame-selection of nucleotide sequences into protein sequences; (3) functional annotation of protein sequences with Pfam^292^ protein families using HMMER 3.3^302^; (4) curation of NCBI Taxonomy^293^ IDs (taxIDs) for MarFERReT candidate entries and additional incorporation of matched IDs and classification from the PR^2^ Taxonomy ecosystem^294,295^; (5) candidate entries are assessed with evidence from external studies and by taxonomic analysis of ribosomal protein sequences for potential cross-contamination. Validated entries accepted for the quality-controlled build are recorded in the entry metadata. (**b**) Quality-controlled MarFERReT build with validated entries. For the set of 800 validated entries, the same methods used in 1a were used for (1) aggregating nucleotide and protein data and (2) translating nucleotide to protein sequences; (3) intra-taxa clustering at the strain or species level: protein sequence data sharing the same NCBI taxIDs are pooled together and clustered^307^ at 99% identity using updated taxIDs contained in the metadata; (4) Final Pfam annotation of the clustered protein sequences; (5) identification of core transcribed genes from functional annotations of transcriptome-derived entries.

A total of 902 candidate reference entries considered for inclusion into MarFERReT were collected online from public-access sequencing projects released between 2002 and 2021, with a predominant focus on marine microbial eukaryotes (Fig. 1a). The reference sequences were gathered from 754 assembled transcriptomes^12,14,17–53^ (including 2 single-cell amplified transcriptomes), and 148 genomes (including 8 single-cell amplified genomes)^54–291^. Sequences were collected from sources as nucleotides or translated peptides. Predicted protein sequences were annotated with Pfam 34.0^292^ to provide uniform functional prediction. Each reference entry is linked to an NCBI Taxonomy^293^ identifier (NCBI taxID) reflecting the latest changes in the NCBI Taxonomy. For 885 candidate entries we have also included matching identifiers from the PR^2^ database ecosystem^294,295^ of protist ribosomal reference sequences. The PR^2^ identifiers^294^ and the addition of 9 classification levels from the community curated PR^2^ database^295^ complement the NCBI Taxonomy classifications, and facilitate cross-comparison with 18 S RNA metabarcoding studies. We use NCBI taxIDs as the primary taxonomic identifier in most of our downstream processes owing to its widespread community adoption, interoperability with last common ancestry (LCA) taxonomic annotation software, and strain-level taxonomic resolution.

**Fig. 2:**
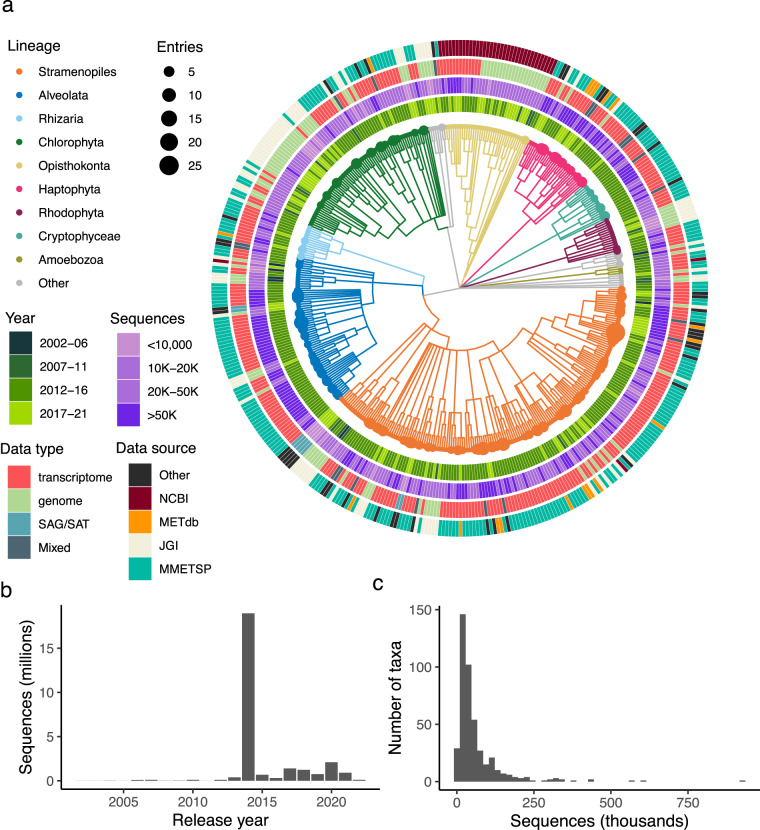
Cladogram of 800 validated MarFERReT entries and summary of reference metadata. (**a**) Cladogram of hierarchical taxonomic ranks of marine eukaryotes within the NCBI Taxonomy framework^293^ using the NCBI CommonTree tool. Each tip is a unique taxon included in MarFERReT, defined by its NCBI taxID identifier. Branches are colored by taxonomic lineage with size of the closed circle at each tip proportional to the number of validated entries in each taxon. Concentric rings describe metadata and statistics for each taxon. From innermost ring outward: year of publication or data release for sequence data (average year of release for multiple entries), number of clustered sequences in taxon, raw input format of sequence data: transcriptome, transcriptome shotgun assembly; genome, genome-derived gene models; SAG, single-cell amplified genome; SAT, single-cell amplified transcriptome; or a combination of types (mixed), and source of data: NCBI^293^, METdb^7^, JGI PhycoCosm^300^, or MMETSP^3^. (**b**) Number of clustered sequences in MarFERReT build by year of data release, and (**c**) Histogram showing distribution of clustered sequence count for 453 taxa in the final build.

We include quality control steps to validate candidate entries for inclusion into a final, quality-controlled library build. An analyses of ribosomal protein taxonomies was applied to all MarFERReT candidate entries to assess sequence data for potential cross-contamination. We also incorporated cross-contamination analyses of MMETSP transcriptome entries^8^ derived from two previous studies^296,297^ (see Technical Validation section). In total, we identified 102 candidate entries with sample contamination or sequence quality issues. The remaining 800 candidate entries were included in the final MarFERReT v1.1 data products^298^ (Fig. 1b), encompassing 453 species or strain-level taxa. Sequence redundancy was reduced within each taxon by combining sequences with the same NCBI taxID and clustering them at a 99% protein sequence identity threshold, resulting in a total of 27,951,013 translated and clustered protein sequences (Fig. 2, see Data Records for full description of data).

The implementation of this workflow consists of a combination of Python, Bash, and R scripts, and is freely available as part of the MarFERReT repository. All core database processing steps have been containerized to maximize reproducibility, reduce issues related to software dependencies, and lower technical barriers to use. Documentation included in the repository details the steps required for users to replicate the construction of MarFERReT or to build derivative versions, as well as example use cases. Additionally, supporting code infrastructure is designed to facilitate the addition of new curated reference sequence material and annotation products by users and in future releases. The project repository containing code and documentation can be found here^299^. MarFERReT v1.1 data products are available online through the Zenodo repository (link)^298^, and contain all data files necessary to begin using MarFERReT for sequence annotation (see Data Records and Usage Notes sections for more detail).

## Methods

### Collation of sequence data

First (arrow 1, Fig. 1a), candidate entries were collected from publicly available web resources in the form of genomic, transcriptomic, and single-cell-amplified genome (SAG) and transcriptome (SAT) data. Reference sequences were collected from primary sources as either nucleotides (5,986,735 sequences) or translated peptides (30,826,074 sequences). Four projects were major sources of sequence information for MarFERReT. The Marine Microbial Eukaryote Sequence Project^3^ (MMETSP) is the largest contributor to MarFERReT with 678 candidate entries. The MMETSP sequences were collected as peptide translations from Version 2 of the MMETSP re-assemblies^8,17^. A total of 116 entries^54–252^ were collected from JGI Phycocosm^300^ as translated protein predictions of gene models from assembled genomes. A total of 41 transcriptomes from Roscoff Culture Collection^18^ isolates were collected from the METdb database^7^ as nucleotide transcriptome assemblies. To provide classification breadth to metazoan (animal) sequences consistently captured in protist-focused metatranscriptomes^2^, sequences from 36 Metazoan species were included from NCBI GenBank, including nucleotide transcriptome assemblies from 14 copepod transcriptomes^26–47^ and translated gene models from 22 marine species selected for broad coverage of 12 metazoan phyla^254–292^. The remaining 23 candidate entries were collected from other sequencing projects including translated gene models from 8 single-cell amplified genomes of uncultured stramenopiles^12^, ten assembled transcriptomes of diatom isolates from Antarctic and North Atlantic coastal waters^13^, two single-cell amplified assembled transcriptomes from early-branching dinoflagellates^14^, and translated transcriptome assemblies from open-ocean isolates of haptophytes and diatoms of the North Pacific^15,25^. Information for each candidate entry is available in the data repository in MarFERReT.v1.metadata.csv^298^ (see the Data Records section), including the web link to the primary data, the original file name, and the associated publication and DOI (link).

### Six-frame translation and frame selection

Second (arrow 2, Fig. 1a), nucleotide sequences were translated in six frames with transeq vEMBOSS:6.6.0.059^301^ using Standard Genetic Code, to bring all reference sequence material into translated amino acid sequence space. The translation frame containing the longest open reading frame sequence was retained for downstream analysis.

### Functional annotation of protein sequences

Third (arrow 3, Fig. 1a), candidate entry protein sequences were annotated against the Pfam 34.0^292^ collection of 19,179 protein family Hidden Markov Models (HMMs) using HMMER 3.3^302^. The highest-stringency cutoff score (‘trusted cutoff’) assigned by Pfam to each hmm profile was used as a minimum score threshold. The best scoring Pfam annotation (highest bitscore) was used if the protein received more than one Pfam match. A total of 12,554,711 candidate entry sequences received annotation with 12,549 of the 19,179 total possible profiles in Pfam 34.0. The raw Pfam annotations and best-scoring annotations are available online in the MarFERReT Zenodo repository (see Data Records).

### Curation of sequence metadata

Fourth (arrow 4, Fig. 1a), NCBI Taxonomy^293^ hierarchical relationships were determined for the NCBI taxIDs in MarFERReT using the ‘taxtastic’ package^303^ v0.9.2. Prior to this, an NCBI taxID for each entry was determined through source metadata or manually assigned. All entries were manually updated if necessary to reflect changes and additions to the NCBI Taxonomy database, as of October 11^th^, 2022. For 761 of the 902 candidate entries; an NCBI Taxonomy was provided with the source metadata. The original taxIDs were unchanged for 497 entries, and for 264 entries, the taxID was updated to reflect the best-possible taxonomic match in NCBI Taxonomy as of October 11^th^, 2022. For the remaining 141 entries without an NCBI taxID from the data source, organism name and strain information were used to find the most specific match in NCBI Taxonomy, searching for strain-specific matches, then species-specific matches if strain was not available, followed by unclassified species in the genus. The complete record of taxID curation for every entry is available online (see ‘MarFERReT.v1.entry_curation.csv’ in Data Records).

Each entry is also associated with its closest match within the PR^2^ protist ribosomal reference database^294^ and the accompanying community-curated classification scheme of the PR^2^ database ecosystem^295^. A custom python script was used with the updated NCBI taxIDs associated with MarFERReT entries to identify the 18 S sequence tags in the PR^2^ database that share the same NCBI taxID. We used RapidFuzz^304^, a rapid fuzzy string matching algorithm to help identify the PR^2^ sequence tags matching MarFERReT entries at the strain-level wherever possible, as the strain identities are not always captured in the NCBI Taxonomy framework and the PR^2^ taxonomy levels only descend to the species level, even if strain information is included in the ID description. Each entry was manually reviewed to identify the most accurate strain-level match, proceeding to species-level matches if no strain match was found, and genus-level if no species-level match exists in the PR^2^ database. The associated PR^2^ ID and the full PR^2^ lineage classification are also available in the entry metadata on Zenodo^298^ (‘MarFERReT.v1.metadata.csv’ in the Data Records section), code documentation is available here (link). For subsequent steps, we use the NCBI taxID for strain-level identification and interoperability with key software.

### Validation of candidate entries

**Fig. 3:**
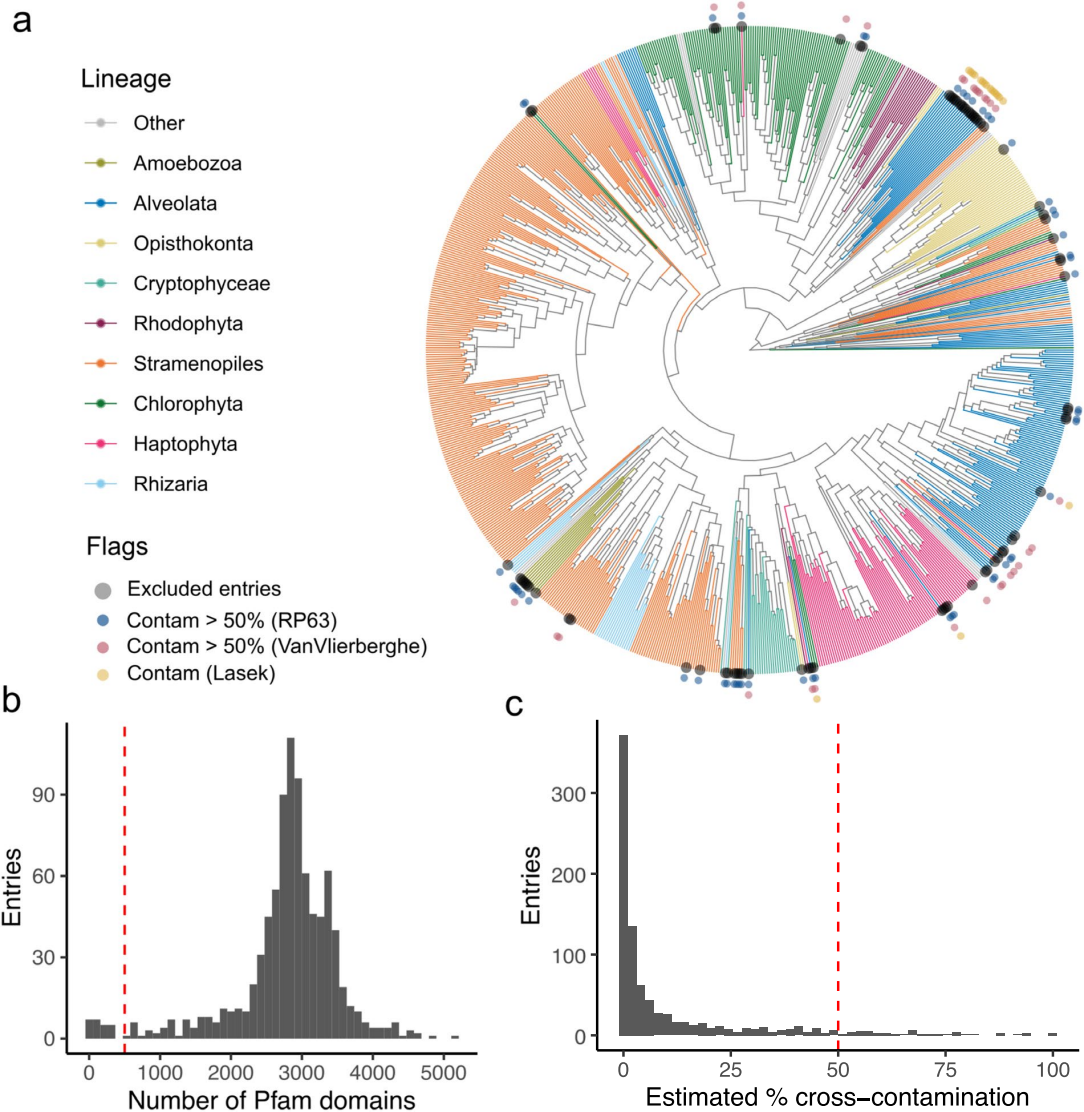
Validation of candidate entry sequences for cross-contamination. (**a**) Circular tree from hierarchical clustering of a binary distance matrix, constructed from the presence/absence of approximately 12,000 Pfam protein families in 874 candidate entries; entries with low sequence or pfam flags were excluded. Grey points at the tip indicate one of 102 entries excluded from the final build, with the other points marking the flag(s) for excluded entries. Contam > 50% (RP63), cross-contamination estimates over 50% from this study; Contam > 50% (VanVlierberghe), cross-contamination estimates over 50% from van Vlierberghe *et al*.^297^; Contam (Lasek), reported contamination for ciliate entries from Lasek-Nesselquist and Johnson^296^. (**b**) Histogram of Pfam domains in annotated candidate entry sequences; red dotted line indicates the 500 Pfam minimum threshold for inclusion. (**c**) Histogram of estimated cross-contamination in entries from ribosomal protein analysis; red dotted line indicates 50% cutoff threshold for exclusion.

Fifth (arrow 5, Fig. 1a), we assessed the 902 candidate entries for potential quality and contamination issues to identify a set of validated entries to include in a quality-controlled MarFERReT build (Fig. 3). Five metrics were used to flag candidate entries for exclusion from the final build. First, we flagged 24 entries with less than 1,200 total sequences, and second we flagged an additional 4 entries with less than 500 total assigned Pfam domains (Fig. 3b) from the functional annotation step. For candidate entries from the MMETSP re-assemblies^8,17^, we incorporated cross-contamination estimates derived from two independent studies. Lasek-Nesselquist and Johnson^296^ examined 26 ciliate MMETSP entries for potential contamination and identified 18 samples with an estimated 25 to 86% contamination; these 18 MMETSP entries were flagged in our assessment. Van Vlierberghe *et al*.^297^ investigated all 678 MMETSP entries for potential cross-contamination through taxonomic analysis of ribosomal protein sequences; we flagged 30 entries with over 50% sequence cross-contamination reported here. The estimates from van Vlierberghe *et al*.^297^ for 8 entries with 100% reported contamination were not flagged as the wrong NCBI taxID was used in their determination (entry IDs 378, 379, 380, 381, 504, 505, 506 and 507).

For the fifth metric, we calculated potential cross-contamination estimates for all candidate entries (Fig. 3c) by deploying an approach similar to the method used to assess MMETSP transcriptomes by van Vlierberghe *et al*.^297^. These estimates were calculated for the 874 candidate entries without the low sequence or low Pfam flags noted above. The approach utilizes taxonomic annotation of ribosomal sequences matched to one of 63 Pfam protein domain IDs specific to ribosomal proteins and present in over 90% of candidate entries (referred to in this manuscript as ‘RP63’). The full set of UniProtKB reference sequences associated with each Pfam domain^305^ was downloaded and used to create a DIAMOND reference database for the DIAMOND^306^ sequence alignment software using the ‘diamond makedb’ command with default parameters and the NCBI database, as scripted in build_diamond_db.sh. Ribosomal protein sequences from MarFERReT candidate entries were retrieved based on Pfam functional annotations (arrow 3, Fig. 1a) and taxonomically identified by Last Common Ancestor estimation using the ‘diamond blastp’ command (parameters: -b 100 -c 1 -e 1e-5 –top 10 -f 102), with results reported as an NCBI taxID. For each entry, we determined the number of ribosomal proteins with an annotation within the pre-specified lineages used by van Vlierberghe *et al*.^297^: Amoebozoa, Ciliophora, Colpodellida, Cryptophyceae, Dinophyceae, Euglenozoa, Glaucocystophyceae, Haptophyta, Heterolobosea, Palpitomonas, Perkinsozoa, Rhizaria, Rhodophyta, Stramenopiles, and Viridiplantae. We also include the broad taxonomic lineages of Metazoa, Bacteria, Archaea, and Viruses to identify potential contamination by these groups. An estimate of potential contamination for each entry was generated by calculating the percentage of ribosomal sequences identified outside of the expected lineage. Based on this analysis, we flagged 53 entries with over 50% estimated contamination (Fig. 3c). These RP63 results are provided in the ‘MarFERReT.v1.QC_estimates.csv’ file described in the Data Records section.

A total of 102 entries failed at least one of the five quality control metrics described above. The metrics and flags are provided in the file MarFERReT.v1.curation.csv as described in the Data Records section. Python scripts and documentation for these analyses are available in the code repository here: (link).

### Final build of MarFERReT with accepted candidate entries

A total of 800 validated candidate entries were accepted for inclusion into the final MarFERReT build, after excluding 102 entries with flags identified during the entry validation step. From these validated entries, protein sequences sharing the same NCBI taxIDs were combined into bins, thus preserving strain-level sequence diversity where possible. Sequence redundancy was reduced (arrow 3, Fig. 1b) by pooling protein sequences within NCBI taxID bins and clustering at the 99% amino acid sequence identity threshold with MMseqs. 2^307^. A representative sequence was retained for each cluster, and all sequences were renamed with a unique identifier. Pfam functional annotations for the 27,951,013 intra-species clustered protein sequences were propagated from the functional annotation step of the validation protocol (arrow 4, Fig. 1b). Clustered protein sequences, taxonomy and entry data tables, Pfam annotations, and other sequence-level protein data are available online (see Data Records for description of all files).

### Identification of Core Transcribed Genes

The Pfam annotations of MarFERReT protein sequences were used to identify a set of cross-taxa core transcribed genes (CTGs; arrow 5, Fig. 1b) that serve as corollary of the BUSCO genome completeness metric^308^ oriented towards marine eukaryotic metatranscriptomes. For any given high-level taxonomic lineage, the CTGs are operationally defined here as the set of Pfam families observed in translated transcriptomes of at least 95% of the species within the given lineage. Only validated entries were used for this analysis. The CTG inventories were identified based on the Pfam 34.0 annotation of 7,514,355 proteins translated from the 654 validated transcriptome and SAT entries (the 146 validated genomic and SAG-sourced entries were not included), and a presence-absence matrix of Pfam functions was generated from the functional annotations of 332 taxa. We derived CTG inventories for all eukaryotes as a whole group, and for nine major marine lineages with at least 10 transcriptomic reference taxa each: Bacillariophyta (diatoms), Ochrophyta (excluding the Bacillariophyta subclade), Dinophyceae, Chlorophyta, Haptophyta, Cryptophyceae, Opisthokonta, Rhizaria and Amoebozoa. Bacillariophyta are listed separately from the Ochrophyta parent clade because this is a well-studied ochrophyte lineage with a large number of sequenced taxa. As an example, the ‘Haptophyta’ CTGs are comprised of the set of 1,074 Pfam domains observed in at least 28 of the 29 haptophyte taxa with transcriptomes in MarFERReT v1.1. The list of Pfam IDs and their detection frequencies are available online for these 9 major marine lineages and for Eukaryotes as a whole^298^ (see the Data Records section, MarFERReT.core_genes.v1.csv), and documented in the code repository (link)^299^.

## Data Records

The final MarFERReT v1.1 data products are available online through a Zenodo repository (link)^298^ and contain the data files necessary to begin using MarFERReT for sequence annotation. The raw source data for the 902 candidate entries considered for MarFERReT v1.1, including the 800 QC validated entries, are available for download from their respective online repositories. Links to the entry source website and associated publications for the entries is listed in MarFERReT.v1.metadata.csv on Zenodo^298^, and detailed instructions and code for downloading the raw sequence data from source are available in the MarFERReT code repository^299^ (link). Sequence source URL locations and linked publications are also available. The permanent DOI representing all MarFERReT versions is 10.5281/zenodo.7055911.

The following files are available in the MarFERReT data repository:

### MarFERReT.v1.metadata.csv

This CSV file contains descriptors of each of the 902 database entries, including data source, taxonomy, and sequence descriptors. Data fields are as follows:

**entry_id**: Unique MarFERReT sequence entry identifier

**accepted:** Acceptance into the final MarFERReT build (Y/N). The Y/N values can be adjusted to customize the final build output according to user-specific needs

**marferret_name**: A human and machine friendly string derived from the NCBI Taxonomy^293^ organism name; maintaining strain-level designation wherever possible

**tax_id**: The NCBI Taxonomy ID (taxID)

**pr2_accession**: Best-matching PR^2^ accession ID^294^ associated with entry (see *Methods*)

**pr2_rank**: The lowest shared rank between the entry and the pr2_accession

**pr2_taxonomy**: PR^2^ Taxonomy classification^295^ scheme of the pr2_accession

**data_type**: Type of sequence data; transcriptome shotgun assemblies (TSA), gene models from assembled genomes (genome), and single-cell amplified genomes (SAG) or transcriptomes (SAT)

**data_source**: Online origin of sequence data; from a Zenodo data repository (Zenodo), a datadryad.org repository (datadryad.org), MMETSP re-assemblies on Zenodo (MMETSP)^17^, NCBI GenBank (NCBI), JGI Phycocosm (JGI-Phycocosm), the TARA Oceans portal on Genoscope (TARA), or entries from the Roscoff Culture Collection through the METdb database repository (METdb)

**source_link**: URL where the original sequence data and/or metadata was collected

**pub_year**: Year of data release or publication of linked reference

**ref_link**: Pubmed URL directs to the published reference for entry, if available

**ref_doi**: DOI of entry data from source, if available

**source_filename**: Name of the original sequence file name from the data source

**seq_type**: Entry sequence data retrieved in nucleotide (nt) or amino acid (aa) alphabets

**n_seqs_raw**: Number of sequences in the original sequence file

**source_name:** Full organism name from entry source

**original_taxID**: Original NCBI taxID from entry data source metadata, if available

**alias:** Additional identifiers for the entry, if available

### MarFERReT.v1.entry_curation.csv

This CSV file contains curation and quality-control information on the 902 candidate entries considered for incorporation into MarFERReT v1.1, including curated NCBI Taxonomy IDs and entry validation statistics. Data fields are as follows:

**entry_id:** Unique MarFERReT sequence entry identifier

**marferret_name:** Organism name in human and machine friendly format, including additional NCBI taxonomy strain identifiers if available

**tax_id**: Verified NCBI taxID used in MarFERReT

**taxID_status**: Status of the final NCBI taxID (Assigned, Updated, or Unchanged)

**taxID_notes**: Notes on the original_taxID

**n_seqs_raw**: Number of sequences in the original sequence file

**n_pfams**: Number of Pfam domains identified in protein sequences

**qc_flag**: Early validation quality control flags for the following: LOW_SEQS; less than 1,200 raw sequences; LOW_PFAMS; less than 500 Pfam domain annotations

**flag_Lasek**: Flag notes from Lasek-Nesselquist and Johnson^296^; contains the flag ‘FLAG_LASEK’ indicating ciliate samples reported as contaminated in this study

**VV_contam_pct**: Estimated contamination reported for MMETSP entries in van Vlierberghe *et al*.^297^

**flag_VanVlierberghe:** Flag for a high level of estimated contamination, from ‘flag_VanVlierberghe’ values over 50%: FLAG_VV

**rp63_npfams**: Number of ribosomal protein Pfam domains out of 63 total

**rp63_contam_pct**: Percent of total ribosomal protein sequences with an inferred taxonomic identity in any lineage other than the recorded identity, as described in the Technical Validation section from analysis of 63 Pfam ribosomal protein domains

**flag_rp63**: Flag for a high level of estimated contamination, from ‘rp63_contam_pct’ values over 50%: FLAG_RP63

**flag_sum:** Count of the number of flag columns (‘qc_flag’, ‘flag_Lasek’, ‘flag_VanVlierberghe’, and ‘flag_rp63’). All entries with one or more flags are nominally rejected (‘accepted’ = N); entries without any flags are validated and accepted (‘accepted’ = Y)

**accepted:** Acceptance into the final MarFERReT build (Y or N)

### MarFERReT.v1.RP63_QC_estimates.csv

This CSV file contains the results of the ‘RP63’ cross-contamination check using ribosomal proteins (see Methods). The lineage bin columns are the taxonomic categories that define whether a query sequence is placed within or outside the expected lineage.

**entry_handle**: Human-readable tag concatenating the MarFERReT ‘entry_id’ with the ‘marferret_name’ (from MarFERReT.v1.metadata.csv)

**entry_id**: Unique MarFERReT sequence entry identifier

**tax_id:** The NCBI Taxonomy ID (taxID)

**n_seqs**: Number of protein sequences annotated as a Pfam ribosomal protein family

**n_pfams**: Number of unique Pfam protein families

**tax_group**: The expected lineage of this entry sample from the ‘predefined lineage’ categories below

**contam_pct**: The percentage of ribosomal protein sequences identified in a lineage other than the expected ‘tax_group’ lineage

**[lineage bins]**: Series of 21 columns Amoebozoa, Ciliophora, Colpodellida, Cryptophyceae, Dinophyceae, Euglenozoa, Glaucocystophyceae, Haptophyta, Heterolobosea, Opisthokonta, Palpitomonas, Perkinsozoa, Rhizaria, Rhodophyta, Stramenopiles, Viridiplantae, Bacteria, Archaea, Viruses, Other, Unknown

### MarFERReT.v1.proteins.faa.gz

This Gzip-compressed FASTA file contains the 27,951,013 final translated and clustered protein sequences for all 800 accepted MarFERReT entries. The FASTA sequence definition line (‘defline’) contains the unique identifier for the sequence and its reference (mftX, where ‘X’ is a ten-digit integer value).

### MarFERReT.v1.taxonomies.tab.gz

This Gzip-compressed tab-separated file is formatted for interoperability with the DIAMOND^306^ protein alignment tool commonly used for downstream analyses (see Usage Notes) and contains some columns without any data. Each row contains an entry for one of the MarFERReT protein sequences in MarFERReT.v1.proteins.faa.gz. Note that ‘accession.version’ and ‘taxid’ are populated columns while ‘accession’ and ‘gi’ have NA values; the latter columns are required for back-compatibility as input for the DIAMOND alignment software and LCA analysis.

The columns in this file contain the following information:

**accession**: (NA)

**accession.version**: The unique MarFERReT sequence identifier (‘mftX’)

**taxid**: The NCBI Taxonomy ID associated with this reference sequence

**gi**: (NA)

### MarFERReT.v1.proteins_info.tab.gz

This Gzip-compressed tab-separated file contains a row for each final MarFERReT protein sequence with the following columns:

**aa_id**: The unique identifier for each MarFERReT protein sequence

**entry_id**: The unique numeric identifier for each MarFERReT entry

**source_defline**: The original, unformatted sequence identifier

### MarFERReT.candidate_entry_Pfam_annotations.tar.gz

This Gzip-compressed archive contains the raw HMMER3^302^ output from the search of Pfam 34.0^292^ HMM profiles against the full set of protein sequences from candidate entries. The archive contains files for each entry with the suffix ‘.Pfam34.domtblout.tab’ and prefixed with the ‘entry_id’ and ‘marferret_name’ values from MarFERReT.v1.metadata.csv. The ‘domtblout.tab’ files are the output from hmmsearch using the–domtblout parameter containing 3 header and 10 footer rows beginning with ‘#’ and rows for each hmmsearch match with 22 whitespace-delimited fields and a target sequence description (see here for more information on the hmmsearch output file formats). The ‘target name’ (original sequence identifier from MarFERReT.v1.proteins_info.tab.gz), ‘query name’ (Pfam name), ‘accession’ (Pfam ID), ‘E-value’ and ‘score’ (full sequence match scores) are retained in downstream data products.

### MarFERReT.v1.best_pfam.csv.gz

This Gzip-compressed CSV file contains the best-scoring Pfam annotation for intra-species clustered protein sequences from the 800 final MarFERReT entries; derived from the raw hmmsearch annotations in MarFERReT.candidate_entry_Pfam_annotations.tar.gz. This file contains the following fields:

**aa_id**: The unique MarFERReT protein sequence ID (‘mftX’)

**entry_id:** Unique MarFERReT sequence entry identifier

**source_defline:** Original FASTA sequence identifier

**pfam_name**: The shorthand Pfam protein family name

**pfam_id**: The Pfam identifier

### MarFERReT.v1.entry_pfam_sums.csv.gz

This Gzip-compressed CSV file contains a reduced version of MarFERReT.v1.best_pfam.csv.gz; grouped by ‘entry_id’ and ‘pfam_id’ to summarize the number of sequences (‘n_seqs’) with each unique entry_id-pfam_id pair. Contains the ‘entry_id’, ‘pfam_id’, ‘pfam_name’ and ‘n_seqs’ columns.

### MarFERReT.v1.core_genes.csv

This CSV file contains the core transcribed gene (CTG) catalog derived from MarFERReT transcribed reference sequence data (see Methods) to be used in environmental metatranscriptome analysis in conjunction with other MarFERReT data products. The columns contain the following values:

**lineage**: Name of major marine microbial eukaryote lineage

**n_taxa**: Number of species- and strain-level taxa this Pfam observed in

**pfam_id**: Pfam protein family identifier

**frequency**: Proportion of species (n_species) in lineage where pfam_id is observed

## Technical Validation

MarFERReT was developed for the primary application of marine metatranscriptome annotation. We used several independent criteria to assess the 902 candidate genome and transcriptome entries from different sources, and flagged 102 entries for exclusion (Fig. 3a) and retained 800 validated entries for the quality-controlled build as detailed in the Methods subsection, *Validation of candidate entries* (Fig. 1a). These flags and related metrics are reported for entries in MarFERReT.v1.entry_curation.csv (see Data Records section).

The ‘LOW_SEQS’ flag was given to 24 transcriptome entries from the MMETSP dataset with less than 1,200 sequences; these low counts were also noted by van Vlierberghe *et al*.^297^. This threshold cutoff is far less than the median sequence count of approximately 31,000 across all entries. We assessed the number of Pfam annotations to candidate entry sequences to ensure that entries properly contained protein-coding sequence content. Over 90% of entries have between 1,500 and 4,500 Pfam domains, and we assigned the ‘LOW_PFAMS’ flag to 4 entries with less than 500 Pfams if they were not already flagged with ‘LOW_SEQS’ (Fig. 3c).

We investigated entries for potential sequence cross-contamination through taxonomic analysis of 63 ribosomal protein sequences (referred to here as ‘RP63’), flagging 53 entries with over 50% estimated cross-contamination (Fig. 3c). This approach is similar to the one described by van Vlierberghe *et al*.^297^, except that it utilizes sequences from the publicly-available Pfam resource^305^ and DIAMOND^306^ Lowest Common Ancestor analysis instead of manually-curated alignments to provide expanded representation, and also identifies entries with cross-kingdom contamination from bacteria (see MarFERReT.v1.QC_estimates.csv in Data Records). The RP63 flags were added to 30 entries with over 50% contamination from van Vlierberghe *et al*.^297^ for the MMETSP transcriptomes and for 18 ciliate MMETSP entries reported as contaminated from Lasek-Nesselquist & Johnson^296^.

Together, we flagged a total of 102 candidate entries (Fig. 3) with sequence quality issues or high levels of potential contamination, and these are not included in the final MarFERReT build (Fig. 2). We recognize that predetermined value cutoffs for entry inclusion have the potential to eliminate valuable and diverse entries from the final product. The Usage Notes section describes how users can create a derivative version of the database by adding or removing entries included in the build process. The final build of MarFERReT v1 data products includes 800 validated MarFERReT entries that encompass 453 taxa and approximately 30 million clustered protein sequences (Fig. 2).

## Usage Notes

The code for reproducing MarFERReT data products from primary source sequence is available in a public repository^299^, along with documentation. The software used for critical database assembly steps is packaged in stable, version-controlled containers, and scripts for pulling these containers from public repositories are included in the repository. Users can make use of pre-built MarFERReT v1.1 data products^298^ or create their own development version of MarFERReT using the containerized workflow described below. Code, documentation, and tutorials for this project are available on the MarFERReT repository: https://github.com/armbrustlab/marferret.

To use the processed MarFERReT protein data and associated data products directly, proceed to step 1 below (*Using MarFERReT data products directly)*. To replicate the MarFERReT build process or create a derivative reference library with the containerized pipeline using the raw source data, skip to step 2 below (*Cloning the MarFERReT repository*).

### Using MarFERReT data products directly

Finalized MarFERReT data products include over 27 million intra-species clustered protein sequences, metadata with curated taxonomy identifiers, Pfam protein annotations, core transcribed gene catalogs for marine microbial eukaryote lineages, and other supporting data^298^. The URLs are provided for the sources of the individual sequences, and the compiled, translated, and clustered sequences are available for download through the public repository, Zenodo (link).

If downloaded directly, steps 2 (*Cloning the MarFERReT repository*) through 7 (*Building the Core Transcribed Gene catalog)* can be skipped. MarFERReT can be combined with other protein sequence reference libraries or new reference sequence material for expanded phylogenetic coverage. For an example of combining MarFERReT with other databases or new reference sequence entries, see step 8 (*Combining MarFERReT with other reference sequences)*. An example workflow for using these data to annotate environmental metatranscriptome is described in subsection 9 below (*Using MarFERReT to annotate environmental metatranscriptomes*).

### Cloning the MarFERReT repository

The first step is to copy the MarFERReT pipeline code and cloning the repository into a suitable directory where the database will be built.

### Collecting and organizing inputs

We do not host the primary raw data in the final MarFERReT data products; the original links, raw filenames, and other source reference information for all candidate entries are available in the MarFERReT entry curation table on Zenodo^298^ (MarFERReT.v1.entry_curation.csv). Two sets of input files are required to replicate the MarFERReT build: 1) the source reference sequences and 2) a corresponding metadata file (MarFERReT.v1.metadata.csv). The source reference sequences will need to be downloaded from their various public locations, and their file names will need to match the entries in the metadata table. The metadata file entitled MarFERReT.v1.metadata.csv contains information on the source data public URL, original file names, URL link to associated publication, and associated data object DOI for all reference sources considered for use in the first MarFERReT build. The ‘accepted’ field indicates entries that were accepted or excluded from the final quality-controlled version of the protein sequence library. These values are given here as a default suggestion and can be toggled to include (“Y”) or exclude (“N”) individual entries to meet the needs of specific research questions. See the full description of this file under Data Records. Detailed instructions for finding and downloading the source reference sequences used to build MarFERReT v1.1 can be found in this document. Once the MarFERReT repository is cloned onto the target machine, a new directory called ‘source_seqs’ must be created under the data directory. All FASTA files of the source reference sequences should be deposited into this directory. Before running the MarFERReT pipeline, all FASTA files should be unzipped.

### Building software containers

The MarFERReT database construction pipeline is containerized to obviate concerns with software dependencies. Additionally, MarFERReT supports both Singularity and Docker containerization, depending on user preference. The necessary containers can be built in two steps:

1. Install either Singularity or Docker on target machine.
2. Navigate to the containers directory and run either the build_singularity_images.sh or build_docker_images.sh script from the command line.

### Running MarFERReT database construction pipeline

Once the input source reference sequences have been collected, metadata has been organized, and the software containers have been built, the MarFERReT database construction pipeline is ready. Navigate to the scripts directory and run the assemble_marferret.sh script from the command line. The user will be prompted to enter either 1 or 2 depending on whether Singularity or Docker containerization is used. The pipeline will take several hours to run, depending on individual computer system specifications. When it is done, the following outputs in the data directory will be available:

- MarFERReT.v1.proteins.faa.gz–MarFERReT protein library
- MarFERReT.v1.taxonomies.tab.gz–taxonomy mapping file required as input for building diamond database
- MarFERReT.v1.proteins_info.tab.gz–mapping file connecting each MarFERReT protein to its originating reference sequence
- /aa_seq–directory with translated & standardized amino acid sequences
- /taxid_grouped–directory with amino acid sequences grouped by taxid
- /clustered–directory with amino acid sequences clustered within taxid

The three.gz files listed above can also be downloaded directly from the Zenodo repository^298^.

### Annotating MarFERReT database sequences

Information on the functions of the proteins included in MarFERReT can be added by annotating the sequences with one of the many bioinformatic tools available for functional inference. In this repository we have included a script for annotating the database with Pfam^292^ (now included as a part of the InterPro consortium^305^).

To annotate MarFERReT, first download a copy of the Pfam database of HMM profiles. Make a new directory named ‘pfam’ under the ‘data’ directory. Download into this directory the latest version of Pfam from the Pfam ftp site.

Once the Pfam HMM database has been downloaded, navigate to the ‘scripts’ directory and run the pfam_annotate.sh script from the command line. In addition to the ‘data/pfam/Pfam-A.hmm’ HMM database, this script requires the ‘data/MarFERReT.v1.proteins.faa.gz’ file as an input. The complete set of Pfam annotations can also be found on Zenodo^302^ (see Data Records for full description).

### Building the Core Transcribed Gene catalog

After the MarFERReT protein sequences have been functionally annotated, sets of CTGs can be derived from the RNA-derived data for specific marine lineages or for all eukaryotes. Selecting the Pfam IDs that are present in at least 95% of species of a given lineage allows us to define a set of functions that can be reasonably expected to be found in a relatively complete transcriptome. These CTG catalogs can be used downstream of environmental sequence annotation with MarFERReT to assess the coverage of environmental taxon bins, as demonstrated in Case Study 2. Documentation and code for generating and using the CTG catalogs from Pfam annotations for user-defined lineages are found here (link).

### Combining MarFERReT with other reference sequences

MarFERReT can be combined with other domain-focused reference sequence libraries or new reference sequence transcriptomes and genomes to expand taxonomic coverage. In the Case Studies, we show an example combining MarFERReT with a filtered version of the prokaryote-focused MARMICRODB library^5,309^. Both libraries use NCBI Taxonomy identifiers as their primary classification framework, facilitating compatible annotation approaches. After downloading or building the MarFERReT protein sequence database, bacterial sequences can be downloaded from the MARMICRODB Zenodo repository^309^ and the libraries concatenated together for use in downstream processes.

MarFERReT can also be combined with individual reference sequence transcriptomes and genomes that have just been released, or are not incorporated in current reference libraries, or to add representation for specific research needs. This also requires that every sequence entry has an NCBI Taxonomy identifier. Instruction and code for combining MarFERReT with other large reference libraries like MARMICRODB or with sets of individual reference sequence entries can be found on the codebase repository here: c ombining_marferret_and_other_references.md.

### Using MarFERReT to annotate environmental metatranscriptomes

**Fig. 4:**
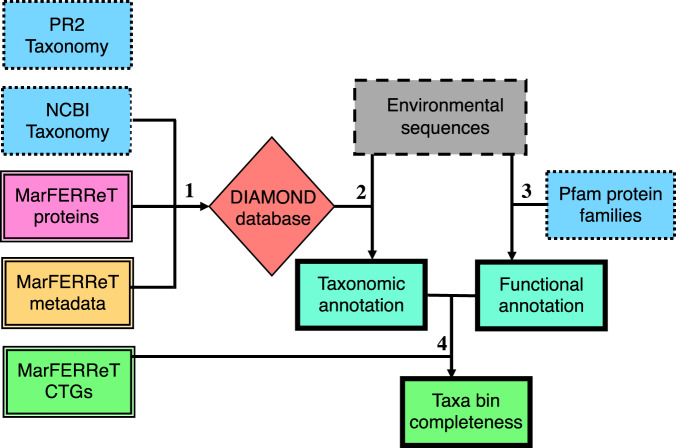
Schematic of case study 1 and 2 use of MarFERReT for annotation of environmental metatranscriptomes. Example workflow showing how MarFERReT data products can be used to annotate unknown assembled sequences and assess taxonomic bins. Boxes indicate datasets; box borders indicate environmental contig sequence data (dashed line), taxonomic and functional annotation from external resources (dotted lines), MarFERReT data products (double lines) and taxonomic and functional annotation results (bold lines). The red diamond indicates a user-constructed DIAMOND^306^ database for lowest common ancestor determination; prior to this step the user could combine MarFERReT with other libraries for expanded taxonomic coverage as shown in case study 1. Arrows represent processes: (1) construction of DIAMOND^306^ database using MarFERReT proteins and data, and taxonomy files from NCBI Taxonomy; matching PR^2^ Taxonomy^294,295^ identifiers and classifications are also provided in the metadata for alternative classification approaches. (2) Taxonomic annotation of environmental contigs using a DIAMOND^306^ database built from MarFERReT proteins; (3) Functional annotation of environmental contigs with HMMER^302^ 3.3 on Pfam^292^ protein family hmm profiles; (4) Completeness assessment of taxonomically- and functionally-annotated metatranscriptome bins using MarFERReT core transcribed genes.

Two case studies, with accompanying code, are provided to illustrate how MarFERReT can be used in a command-line environment for common analyses, either by itself or in conjunction with other protein sequence libraries to assign taxonomic identity to environmental sequences, and to approximate sequencing coverage within environmental taxonomic bins (Fig. 4).

Case Study 1 shows how MarFERReT can be used to annotate unknown environmental sequences using the DIAMOND^303^ fast protein-alignment tool. In summary, a DIAMOND-formatted database is created from sequence data and NCBI Taxonomy^293^ information, and subsequently used to annotate unknown environmental reads (Fig. 4, arrow 1 and 2).

Case Study 2 documents how to identify core transcribed genes for user-defined lineages using MarFERReT protein sequences, and provides an example on how to estimate the completeness of environmental transcriptome bins with taxonomic annotation (from Case Study 1) and functional annotation with Pfam 34.0^292^ (now included as a part of the InterPro consortium). The example shown here uses ‘species-level’ annotations (or lower) for enhanced taxonomic specificity. In summary, the taxonomic and functional annotations are aggregated together, and the percentage of lineage-specific CTGs is determined for each species-level environmental taxon bin (Fig. 4, arrow 3 and 4).

While the case studies discussed above demonstrate the practicality of using MarFERReT for annotating environmental metatranscriptomes, MarFERReT can also be used to supplement other analyses that depend on reference proteins, including the analysis of marine metagenomes and metaproteomes.

### Future MarFERReT releases

MarFERReT was designed to be updated as new microbial eukaryote functional reference sequences are publicly released, with releases identified either through literature reviews, updates to public repositories, or through user nominations. Additionally, users can create their own database derivatives using this framework. Suggestions for new additions or changes to future versions of MarFERReT can be submitted via the ‘Issues’ request function in this code repository (link). When submitting an organism request for future MarFERReT versions, the following information is required:

- Full scientific name of the organism (with strain name if possible)
- An NCBI taxID of the organism (as specific as possible, e.g. strain-level)
- A URL to the location of the assembled source data, with additional instructions if necessary
- Brief justification for why this organism should be included, e.g. “New SAGs from a clade of marine haptophytes”.
- A citation or publication for the data, if available.

New entries will be processed through the workflow described for candidate entries and validated through phylogenetic analysis of ribosomal protein families (see Methods and Technical Validation). Future versions of MarFERReT will be documented in a changelog in the code repository (link), describing any additions or modifications to the library composition. The changelog will detail updates to the MarFERReT code and MarFERReT files hosted on Zenodo^298^, including revisions to the scripts, metadata files, functional annotation protocols, protein sequence library, DIAMOND databases, and Core Transcribed Gene inventories.

## Data Availability

Code, documentation, and tutorials for this project are available on the MarFERReT repository^299^: https://github.com/armbrustlab/marferret. This repository has also been archived for the v.1.1 release^310^ and the archived code is available on Zenodo here: https://zenodo.org/records/10278540. Information on the software versions and parameters used in this publication are included in the MarFERReT containerized build on the repository and in the archived code.
